# Public-private partnerships in primary health care: a scoping review

**DOI:** 10.1186/s12913-020-05979-9

**Published:** 2021-01-04

**Authors:** Nasrin Joudyian, Leila Doshmangir, Mahdi Mahdavi, Jafar Sadegh Tabrizi, Vladimir Sergeevich Gordeev

**Affiliations:** 1 Tabriz Health Services Management Research Center, Iranian Center of Excellence in Health Management, Tabriz University of Medical Sciences, Tabriz, Iran; 2 Social Determinants of Health Research Center, Health Management and Safety Promotion Research Institute, Tabriz University of Medical Sciences, Tabriz, Iran; 3 Department of Health Policy& Management, School of Management & Medical Informatics, Tabriz University of Medical Sciences, Tabriz, Iran; 4 National Institute of Health Research (NIHR), Tehran University of Medical Sciences, Tehran, Iran; 5 Erasmus School of Health Policy and Management (ESHPM), Erasmus University Rotterdam, Rotterdam, The Netherlands; 6 Institute of Population Health Sciences, Queen Mary University of London, London, UK; 7 Department of Infectious Disease Epidemiology, London School of Hygiene & Tropical Medicine, London, UK

**Keywords:** Public-private partnership, Primary health care, Health policy and system research, Decision-making

## Abstract

**Background:**

The Astana Declaration on Primary Health Care reiterated that PHC is a cornerstone of a sustainable health system for universal health coverage (UHC) and health-related Sustainable Development Goals. It called for governments to give high priority to PHC in partnership with their public and private sector organisations and other stakeholders. Each country has a unique path towards UHC, and different models for public-private partnerships (PPPs) are possible. The goal of this paper is to examine evidence on the use of PPPs in the provision of PHC services, reported challenges and recommendations.

**Methods:**

We systematically reviewed peer-reviewed studies in six databases (ScienceDirect, Ovid Medline, PubMed, Web of Science, Embase, and Scopus) and supplemented it by the search of grey literature. PRISMA reporting guidelines were followed.

**Results:**

Sixty-one studies were included in the final review. Results showed that most PPPs projects were conducted to increase access and to facilitate the provision of prevention and treatment services (i.e., tuberculosis, education and health promotion, malaria, and HIV/AIDS services) for certain target groups. Most projects reported challenges of providing PHC via PPPs in the starting and implementation phases. The reported challenges and recommendations on how to overcome them related to education, management, human resources, financial resources, information, and technology systems aspects.

**Conclusion:**

Despite various challenges, PPPs in PHC can facilitate access to health care services, especially in remote areas. Governments should consider long-term plans and sustainable policies to start PPPs in PHC and should not ignore local needs and context.

## Background

Achieving the highest possible level of health is a fundamental right for every human being [1]. Two years ago, 40 years after signing the Declaration of Alma-Ata (1978) [2], world leaders reinstated that ‘strengthening Primary Health Care (PHC) is the most inclusive, effective and efficient approach to enhance people’s physical and mental health, as well as social well-being’ [3]. The Astana Declaration on PHC (2018) reiterated that PHC is a cornerstone of a sustainable health system for universal health coverage (UHC) and health-related Sustainable Development Goals. It also called for all stakeholders to work as partners while taking joint action to build stronger and sustainable PHC [3]. When implementing this Declaration, countries will choose their unique paths towards UHC. Regardless of their choice, all of them would require effective cooperation and involvement of all major stakeholders (i.e., patients, health professionals, the private sector, civil society, local and international partners, and others).

Previous studies have shown that, despite substantial contributions and previous successes, provision of PHC services solely via the public sector providers has its limitation and some potential problems are well-documented (e.g., shortage of human resources, inefficient institutional frameworks, inadequate quality and efficiency due to a lack of competition, particularly in remote and rural areas) [4, 5]. In response to these challenges, some suggested that public-private partnerships (PPPs) initiatives could help to make PHC services provision more effective and efficient [6–12]. PPPs are voluntary cooperative arrangements between two and more public and private sectors in which all participants agree to work together to achieve a common purpose or undertake a specific task and to share risks and responsibilities, resources and benefits [13]. The flexible nature of PPPs provides a framework for developing and adapting existing structures to meet the specific needs of each project [14]. For instance, among the objectives of PPPs could be the establishment of a sustainable financial system; capacity-building reforms and management reforms in the public and private sectors; preventing unintended outcomes in the growth of the private sector in health; cost control and improving the health of the community; facilitating socio-economic development; improving PHC services coverage, quality, and infrastructure; as well as increasing the demand for health services [15].

Local support and private initiatives could become viable when improving PHC performance under a PPP, particularly in a situation when PHC does not have the necessary facilities to provide services, the utilisation of PHC services provided by the public sector is low, and there is a lack of effective mechanisms to evaluate and monitor its performance [5]. Private providers may also play an important role in the management of public health problems, such as malaria, sexually transmitted diseases, and tuberculosis (TB) [4, 16]. It was previously shown that among the main reasons for service uptake from private PHC providers were better geographic access, shorter waiting times, more flexible opening hours, easier access to staff consultations and medication, and more confidentiality regarding disease-related symptoms [4, 17–20]. Moreover, the use of PPPs can significantly reassure and reduce the fear of privatising health care services [6]. Not surprisingly, PPPs are rapidly expanding and becoming an integral part of effective health interventions [21]. They have been tested as a means of ensuring the provision of comprehensive PHC service is efficient, effective, and fair [22]. PPPs are also often perceived as an innovative method that can produce desired results, particularly when the market fails to distribute health benefits to those who need them (i.e., disadvantaged and the poor people in developing countries) [23, 24].

Our scoping review aimed to examine evidence on the use of PPPs in the provision of PHC services and answer the following questions: What target groups have been assigned to receive PHC services via PPPs? What kind of PHC services and processes were provided via PPPs? What arrangements or methods have been used to transfer PHC services to a private sector? What are the results of the service delivery using PPPs? What is the experience of PHC service users? What were the lessons learnt?

## Methods

### Data sources and search strategy

Six databases (ScienceDirect, Ovid Medline, PubMed, Web of Science, Embase, and Scopus) were searched between September and October 2018 for studies reporting on PPPs models used in PHC services provision. We used the following search terms: PPP or public-private partnership(s), public-private participation, public-private collaboration, public-private engagement, public-private mix, in combination with PHC or primary health care, primary healthcare, health care, healthcare, public health. A detailed search strategy for each database can be found in Appendix. The publication language was restricted to English. There were no time restrictions. We supplemented our review by a grey literature search conducted using the World Health Organization databases and websites of private health institutions. Additionally, the references of all included papers were searched for articles not identified through electronic searches.

### Study selection

The titles and abstracts of documents were assessed against the inclusion and exclusion criteria (Appendix) by two co-authors (NJ and LD). Any disagreements were be resolved by a third independent reviewer (JST). References were managed using EndNote X8 (Thomson Reuters, Philadelphia, PA, USA).

### Data extraction and synthesis

We extracted the following information from the studies included in the review: setting, objectives, type of study, services, type of model, results, challenge, and recommendation (Table 1). Based on extracted data, we identified themes related to challenges, design, and implementation recommendations of PPPs projects in PHC service provision.

**Table 1:** Key features of studies included in the review

Author, year	Location /setting	Objective(s)	Type of study	Services	Model type	Target group
Ahmed F, Nisar N, 2010 [15]	Pakistan	Examines barriers to further development of PPP in Pakistan	Narrative review	–	PPP (contracting out)	–
Ardian M et al., 2007 [13]	Indonesia	To describe a successful partnership between the district health department, a private company and non-governmental health care providers	Descriptive-case study	Case detection, treatment	PPP	TB case
Argaw MD et al., 2016 [25]	Ethiopia	To analyse health facility reports on malaria service delivery to assesses the magnitude of cases and adherence of health care workers on the national standards	Retrospective descriptive	Malaria diagnosis and treatment	PPP	Suspected of malaria
Arora V et al., 2004 [22]	India	To increase case notification in the revised national TB control program and to improve treatment outcome in the private sector through the implementation of dots principles	Intervention	Diagnosis and treatment of TB patients	PPM	Suspected of TB, Patient with TB
Baig M et al., 2014 [6]	India	Assess the nature and extent of primary health care services provided in PHCs managed by NGOs and Corporates, as compared to the government	A case study	Immunisation services, health promotion, treatment of common ailments, malaria management, delivery services	PPP	The people of a region
Balasubramanian R et al., 2006 [26]	India	To evaluate a rural public-private partnership model within the TB control program	Cohort	Case detection, treatment	PPP	TB case
Barr DA, 2007 [27]	USA	Provide an overview of the history of health-related PPPs during the past 20 years and describe a research protocol commissioned by the world health organisation to evaluate the effectiveness of PPPs in a research context	Descriptive protocol	–	PPP	–
Bourgeois DM et al., 2014 [28]	UK	Introducing an oral health collaborative promotion program called Live.Learn.Laugh	Narrative review	Educational and research services of oral health	PPP	Schools, kindergarten, mothers of students, the general population
Brad Schwartz J, Bhushan I, 2004 [7]	Cambodia	To examine the effects on immunisation equity of the large-scale contracting of primary healthcare services in rural areas of Cambodia	Intervention	Coverage targets and equity targets for all primary healthcare services, including immunisation of children	PPP	Five of nine rural districts which together have a population of over 1.25 M people
Chongwe G et al., 2015 [29]	Zambia	Determine the extent of private-sector capacity, participation, practices and adherence to national guidelines in the control of TB	Cross-sectional survey	Diagnose TB- manage a case of TB by providing drugs	PPM	TB case
Dewan PK et al., 2006 [8]	India	To review the characteristics of the PPM projects in India and their effect on case notification and treatment outcomes for TB	Literature review	Diagnosis and treatment of TB patients	PPM	Patients with TB and those suspected to have TB
Ejaz I et al., 2011 [30]	Pakistan	Presenting viewpoints of government, NGOs and donors in Pakistan about PPP	Qualitative	–	PPP	–
Engel N, van Lente H, 2014 [9]	India	Discuss three early PPMs from the point of view organisational innovation and control practices	Qualitative	–	PPM	–
Farahbakhsh M et al., 2012 [23]	Iran	Compare the performance quality of two cohorts of public and cooperative health centres in several health service delivery programs throughout 2001–2002	Cross-sectional comparative	Immunisation, maternal, child healthcare, family planning, environmental health, school health, health education, outpatient visits	PPP (contracting out)	The population of the region (9000 to 17,000)
Fobosi S et al., 2017 [31]	South Africa	To inform future service development for sex workers and describe the North Star’s contribution to healthcare provision to this population in South Africa	Case study	Healthcare service package in roadside wellness clinics	PPP	Truck drivers, sex workers and their clients, and individuals from the surrounding communities that do not otherwise have access to clinics
Ganguly P et al., 2014 [32]	India	To explore the factors influencing private obstetricians’ decisions to enrol in the “Chiranjeevi Yojana” scheme, reasons behind their willingness or reluctance to continue, and the reasons why some choose never to participate at all	Qualitative	Providing free intrapartum care	PPP	Poor and tribal women
Ghanashyam B, 2008 [33]	India	A report on health care status in India and reviewing opinions for entering the private sector and participating in primary care	Descriptive	–	PPP	–
Gidado M, Ejembi C. 2009 [34]	Kaduna state, Nigeria	Comparing the roles of public and private health care facilities in the TB program and TB case management practices and treatment outcomes among patients managed in these health facilities	Comparative cross-sectional	Case detection, treatment	PPM	TB case
Gold J et al., 2012 [35]	Australia	Report of experience of partnering with a large private telecommunications provider in order to deliver a health promotion intervention using mobile phone text messages (SMS)	Descriptive	Send a promotional message to promote sexual health	PPP	Eligible mobile advertising subscribers
Handler AS, et al., 2015 [36]	USA	To illustrate how the Illinois breast and cervical cancer program as the public entity has partnered with private physicians, community clinics, and hospitals to effectively deliver breast and cervical cancer services to low-income women across Illinois	Qualitative	Provide quality screening, promote diagnostic services for early detection of breast and cervical cancer, disseminate culturally sensitive public information and education programs	PPP	Women of low income, racial/ethnic minorities, rarely or never screened, and older women
Harris DM, et al., 2012 [37]	USA	To raise awareness of the use of school salad bars as an important part of a comprehensive public health effort to improve child nutrition, to place 6000 salad bars in schools over 3 years	Perspective	Promote fruit and vegetable consumption among schoolchildren by school salad bars	PPP	School-age youth
Herman NG, 2008 [38]	New York city	Describes the program developed by New York College of Dentistry to improve New York city head start children’s oral health	Descriptive-case study	A comprehensive oral health program (educational, preventive and treatment services)	PPP	Preschool children in low-income families
Hirano D, 1998 [39]	USA	Provides an overview of the Arizona partnership for infant immunisation, a coalition of the Arizona Department of Health Services and its partners in the public and private sectors	Overview	Implementing the Arizona Department of Health Services infant immunisation action plan, improve service delivery, provider awareness, community awareness	PPP	All children 2 years of age by the year 2000
Imtiaz A et al., 2017 [10]	Pakistan	To assess the utilisation of maternal and child health services before and after implementation of PPP in district Abbottabad, Pakistan	A cross-sectional study	Vaccination of children in an expanded program of immunisation, vaccination of women for tetanus toxoid, postnatal visits, family planning	PPP	Maternal and child
Joloba M et al., 2016 [40]	Uganda	Redesigned the TB specimen transport network and trained healthcare workers to improve multidrug-resistant TB detection	Intervention	Improving multidrug-resistant TB detection by TB specimen, transport network development and training, mapping and specimen referral to the national TB reference laboratory	PPP	Patients with TB and those suspected to have TB
Kell K et al., 2018 [11]	UK	Report the main outcomes of the past 12 years of partnership, in particular, the key outreach and figures of phase III evaluation	Descriptive	Phase I: 2005–2009 Multiple objective public health programs; Phase II: 2010–2013 Oral health education and promotion programs with a focus on children, patients, mother and infants, communities; Phase III: 2014–2016 School oral health program,21-days education program and world oral health day activities	PPP	Children, patients, mother and infants, communities
Kim HJ et al., 2009 [41]	Korea	To improve treatment outcomes in the private sector by developing a public-private collaboration model for strengthening health education and case holding activities with public health nursing in the private sector	Prospective cohort study	Diagnosis and treatment of TB patients	PPP	Patients with TB and those suspected to have TB
Kramer K et al., 2017 [42]	Tanzania	To comprehensively describe the functioning of the Tanzanian national voucher scheme and examines the effectiveness and equity of the scheme	Case study	Provide three voucher distribution models to increase the reach of target groups for insecticide-treated nets and long-lasting insecticidal nets	PPP	Pregnant women and infants
Kumar M, et al., 2005 [43]	India	Describe and analyse the outcomes of a pilot project PPP and laboratory-based surveillance	Intervention	Diagnosis, treatment	PPP	TB case
Kumar M, et al., 2016 [44]	India	To assess the function of mobile medical units in Jharkhand, India and to identify the factors influencing the utilisation of mobile medical units	Cross-sectional comparative	Provide curative as well as preventive services	PPP	People in 24 remote and hard-to-reach districts in Jharkhand, India
Loevinsohn B et al., 2005 [45]	USA	Examine the effectiveness of contracting, examine the extent to which anticipated difficulties occurred during implementation, make recommendations about future efforts in contracting	Review	–	PPP (contracting out)	–
Loevinsohn B, et al., 2009 [46]	Pakistan	To evaluate the performance of the contractor, health facility surveys, household surveys, and routinely collected information were used to compare the experimental district with a contiguous and equally poor district	Cross-sectional comparative	Provide a broad range of PHC services, including preventive, promotion and curative care	PPP	The population covered in Rahim Yar Khan, Pakistan
Lönnroth K, et al., 2004 [47]	India, Viet Nam, Kenya, India	To compare processes and outcomes of four PPM projects on dots implementation for tuberculosis control in New Delhi, India; Ho Chi Minh City, Viet Nam; Nairobi, Kenya; and Pune, India	Cross-project analysis	Diagnosis and treatment of TB patients	PPM	Patients with TB and those suspected to have TB
Miles K et al., 2014 [21]	Papua New Guinea	Describes a multifaceted PPP between a major oil and gas producer, the national Department of Health and associated development partners in Papua New Guinea	Descriptive	Providing HIV prevention, education and treatment services	PPP	The local population - HIV-infected people, basic HIV awareness education to church groups, community groups and local female sex workers
Mili D, Mukharjee K. 2014 [5]	India	To understand the reasons for participation of NGOs in the PPP program, gauge the extent of community involvement and the benefits accrued by the communities in this program	Cross-sectional	Provide basic health care services	PPP (contracting out)	The population of the poor and remote areas of Arunachala Pradesh, India
Mohanan M, et al., 2013 [48]	Gujarat, India	To evaluate the effect of the Chiranjeevi Yojana program, a PPP to improve maternal and neonatal health in Gujarat, India	Observational study	Maternity services	PPP	Maternal and neonatal among poor women
Mudyarabikwa O, Regmi K, 2016 [49]	UK	To assess to what extent PPPs would increase efficiency in public procurement of primary healthcare facilities	Qualitative	–	PPP	–
Murthy K, et al., 2001 [20]	India	To determine whether private practitioners and the government can collaborate with a non-governmental intermediary to implement directly observed treatment, short-course strategy (DOTS) effectively	Cross-sectional	Case detection, treatment	PPP	Patients with TB and those suspected to have TB
Newell JN et al., 2004 [50]	Nepal	To implement and evaluate a PPP to deliver the internationally recommended strategy dots for the control of TB in Lalitpur municipality, Nepal	Intervention	Diagnosis and treatment of TB patients	PPP	Patients with TB and those suspected to have TB
Newell JN et al., 2005 [51]	Nepal	To describe leadership, management and technical lessons learnt from the successful implementation of a PPP for TB control in Nepal	Qualitative	–	PPP	–
Njau R et al., 2009 [52]	Tanzania	Extensive literature review of various PPP models in health in scale and in scope which are aimed at advancing public health goals in developing countries	Case study	–	PPP	–
Oluoha C et al., 2014 [12]	Nigeria	To assess the contribution of the private health facilities in providing immunisation services in four local government areas	Retrospective descriptive	Immunisation services	PPP	Children
Pal R, Pal S, 2009 [53]	India	Analyse the progress and success of PHC in the new millennium	Perspective	–	–	–
Pérez-Escamilla R. 2018 [54]	China, India, South Africa, Germany, the United Kingdom, Brazil and Mexico	To identify the key factors of successful implementation of Mondelēz International Foundation-supported school-based PPPs in seven countries	Qualitative	Fostering healthy dietary and physical activity behaviours	PPP	Children, adolescents, women, mothers, pregnant women
Perry CL et al., 2015 [55]	USA	Introduces a special issue of seven articles on childhood obesity from the centre and the implications of this research for obesity prevention	Literature review	To address child health issues through research, service, and education	PPP	Child health
Quy H et al., 2003 [56]	Vietnam	To assess the impact on case detection of a PPM project linking private providers to the National TB Program	Intervention	Case detection. Treatment.	PPM	TB case
Ramiah I, Reich MR, 2006 [57]	Botswana	Analyses the multiple challenges that African Comprehensive HIV/AIDS Partnerships confronted in its first 4 years in the building and managing its relationships with other organisations and among African Comprehensive HIV/AIDS Partnerships partners	Qualitative	–	PPP	–
Rangan S et al., 2004 [58]	India	To develop a ‘model’ partnership between rural private medical practitioners and the revised national TB control program	Intervention	Diagnosis, treatment	PPM	TB case
Reviono R et al., 2017 [59]	Indonesia	To explore the case detection achievements of the tuberculosis program since ppm implementation in central Java, Indonesia in 2003	Retrospective cohort study	Case detection, treatment	PPM	TB case
Ribeiro CA et al., 2016 [60]	Brazil	Describe public-private partnership in the prevention of influenza amongst industry workers in Ceara state, Brazil	Case study	Vaccination against influenza	PPP	Industry workers
Sheikh K et al., 2006 [61]	India	Review two studies on the private sector in India for TB and HIV, and highlight future policy directions for involving PPM in public health programs	Cross-sectional observational	Diagnosis, referrals, TB and AIDS treatment	PPM	Those suspected to have TB and AIDS
Sil et al., 2010 [62]	USA	To improve access to quality oral health care in central Massachusetts with the central Massachusetts oral health initiative	Case study	Oral health care	PPP	Mothers, pregnant women, children
Silow-Carroll S, 2008 [63]	USA	Describe the implementation of Iowa’s 1st Five Initiative: improving early childhood development services through public-private partnerships	Narrative review	Screening referrals and follow-up for mental health	PPP	All young children ages 0 to 5 years and their families
Sinanovic E, Kumaranayake L, 2006 [4]	South Africa	To evaluate the quality of care for the treatment of TB provided in different PPPs	Cross-sectional comparative	Case detection, treatment	PWP, PNP	TB case
Singh A et al., 2009 [64]	India	Investigating the provision of skilled nursing care and emergency care for the poor through a partnership with the private household in Gujarat, India	Observational study	Maternity services	PPP	Poor women
Tanzil S et al., 2014 [65]	Pakistan	Assess the range and quality of healthcare services at the basic health units in Sindh, Pakistan, administered by the district governments as compared to the basic health units which are being contracted and now managed by the peoples’ primary healthcare initiative	Cross-sectional survey	Primary health care services	PPP (contracting out)	The population of the district
Ullah ANZ et al., 2012 [66]	Bangladesh	Analyses the basic concepts and key issues of the existing collaboration between government and NGOs in health care	Qualitative	–	PPP	–
Uplekar M, 2003 [67]	Switzerland	Presents the guiding principles of PPM dots and major elements of the global strategy	Narrative review	–	PPM	–
Uplekar, 2016 [68]	Switzerland	To present a global perspective on the progress and prospects of expanding PPM for TB care and prevention	Perspective	–	PPM	–
van de Vijver S, et al., 2013 [69]	Sub-Saharan Africa	To describe a study design that integrates public health and private-sector approaches to lead to the development and introduction of a service delivery package for cardiovascular disease prevention among urban poor	Descriptive	Awareness Access to screening for CVD risk factors Treatment Seeking Long-term compliance	PPP	Korogocho, a Nairobi slum with a total population of 35,000, screening is for those over 35 years old
Zafar Ullah A, et al., 2006 [70]	Bangladesh	To develop and evaluate a PPP model to involve private medical practitioners in the TB control activities	Intervention	Diagnosis Treatment follow-up	PPP	TB case

*PPPs* Public-Private Partnerships, *TB* Tuberculosis, *NGO* non-governmental organisations, *PPM* Public-Private Mix, *PWP* Public-Private Workplace Partnership, *PNP* Public-NGO Partnership

## Results

**Fig. 1:**
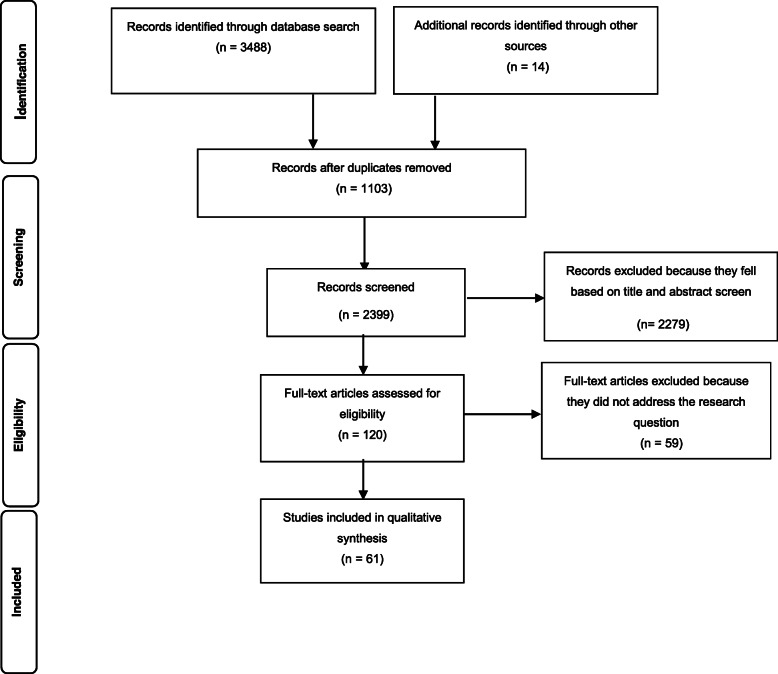
PRISMA flow chart of study selection

The results of the screening process are shown in Fig. 1. In total, 3488 documents were screened by title and abstract for possible inclusion in the review. Additional 14 documents were identified through the manual search. After screening all titles and abstracts, the full text of 120 documents was assessed against the eligibility and exclusion criteria, and 61studies were selected and included in the final review (Table 1). Of 61 selected studies, 32 studies were conducted in Asian countries; 11 studies in African countries; 11 studies in North and South American countries; one study was conducted in several different countries; three studies in the UK; two studies in Switzerland and one study in Australia. Of 61 selected studies, ten (16.3%) were descriptive, nine (14.7%) were qualitative, eight (13.1%) were case studies, eight (13.1%) were intervention studies, eight (13.1%) were reviews, seven (11.5%) were cross-sectional comparative studies, five (8.2%) were cross-sectional studies, three (4.2%) were cohort studies, and three (4.2%) were prospective studies. We observed that reported PPPs fell into one of the three broad categories: PPPs contracted out for basic PHC services, PPPs in health education and promotion programs, and PPPs in services for infectious diseases. Hence, we used this categorisation to summarise our findings.

### PPPs contracted out for basic PHC services

A wide range of basic PHC services in Iran, England, Pakistan, India, Nigeria, Cambodia, Brazil, Arizona (US) and Bangladesh (i.e., prevention, promotion**,** and medical care, including maternal and child health care, family planning, environmental health, school health, health education, immunisation services, health promotion, common diseases treatment, malaria management, maternity services, postpartum services**,** and vaccination against influenza) was outsourced to PPPs and delivered to specific target groups (i.e., children, mothers, pregnant women, industrial workers, poor residents in the remote areas). The provision of these basic PHC services (infrastructure, procurement**,** and services management) was contracted out to private sector providers to facilitate better access and coverage of the population. The majority of studies reported that the provision of basic PHC services by private sector actors increased access to services, improved aspects of care, and resulted in various positive outcomes [5, 7, 10, 12, 23, 33, 39, 44–46, 49, 53, 60, 64, 65]. However, there was also some criticism as well. For example, Baig et al. [6] showed that the management of immunisation services, health promotion, disease treatment, and malaria by PPPs could also be seriously inadequate. Mahan et al. [35] also reported that due to being perceived as having poor quality by the local population, the uptake of institutional and maternal delivery provided in the private hospitals was low despite being offered free of charge.

### PPPs in health education and promotion programs

Studies provided evidence regarding successful PPPs project implementation in the field of health education and promotion (i.e., oral health, sexual health, screening programs, and nutrition). For example, a PPP launched in 2010 by the FDI World Dental Federation and the Unilever measurably improved the oral health among children by encouraging children in kindergartens and schools, student mothers and the general population to brush teeth with a fluoride-containing toothpaste at least twice a day [11, 28]. The comprehensive Oral Health Program created by the New York Dental College [38] for preschool children from low-income families and the Central Massachusetts Oral Health Initiative (CMOHI) [62] in the US for mothers, pregnant women, and children, also increased access to oral health care.

In the field of sexual health in South Africa, the North Star Alliance (a not-for-profit, non-governmental organisation established in 2006) united the transport sector in its response to the AIDS pandemic. It provided healthcare service package in roadside wellness clinics for truck drivers, sex workers, and their clients, as well as individuals from surrounding communities that do not have access to clinics otherwise. They also referred patients with complications to other health facilities in collaboration with the government and other non-governmental organisations [31]. In Australia, a partnership between the research institutions and the telecommunications service provider was established to promote sexual health. The research institutes created the content of the sexual health campaign sent via text messages, while the telecommunications service provider performed randomisation of eligible mobile advertising subscribers for broadcasting the text messages [35].

In the field of breast and cervical cancer screening, in the US, the cooperation of a public institution with a group of private physicians, community clinics and hospitals led to a provision of better quality screening services. These included the provision of updated diagnostic services, the dissemination of educational, cultural and general information to low-income groups, racial and ethnic minorities, and senior women [36]. In South Africa, a package of interventions to prevent cardiovascular diseases among poor citizens was designed and implemented with the participation of the private sector, which had an overall positive reaction of the population [69]. In the field of mental health screening, a successful project was launched in Iowa (the US) through the Commonwealth Fund’s Assuring Better Child Health and Development II project. This project created a coalition of public and private partners that focused on designing, testing, and identifying best practices for enhancing health care providers’ mental health screening and referrals for all young children and their families, and ensuring effective coordination of assessment, intervention, follow-up, and communication back to the primary care practitioners [63].

In the field of nutrition, in 2010, in the US, the National Fruit & Vegetable Alliance, United Fresh Produce Association Foundation, the Food Family Farming Foundation, and the Whole Foods Market together launched the School Salad Bars initiative. This program provided resources and support to schools implementing the School Salad Bars initiative to raise awareness of the use of school salad bars, promote the consumption of fruits and vegetables among school students and improve their nutrition. This initiative resulted in an uptake of this program by approximately 700,000 students in 2012 [37]. Another successful global project was implemented in Asia (China and India), Africa (South Africa), Europe (Germany, United Kingdom**),** and Latin America (Brazil and Mexico). It fostered developing healthy diet habits and promoted the adoption of a physically active lifestyle among children, adolescents, women, mothers, and pregnant women [54].

In the field of health research, a successful PPP in Texas (the US) was established to prevent childhood obesity by focusing research on improvements in children, family, and community health through etiologic, epidemiologic, methodologic, and intervention research [55]. Other successful partnerships (e.g., Medicines for Malaria Venture and Global Alliance for TB Drug Development) have been implemented to facilitate universal access to essential drugs and health services, accelerate research and development in the fields of vaccines, diagnostics, and drugs for neglected diseases [52].

### PPPs in services for infectious diseases

Studies also provided evidence regarding successful PPPs in the delivery of infectious disease services (i.e., malaria, TB, HIV/AIDS). PPPs in malaria case management in Tanzania and Ethiopia led to successful results and increased benefits among pregnant mothers, infants, and patients with malaria [25, 42]. PPPs formed by governments, international donors, and pharmaceutical companies in India and several African countries were also successfully used to control AIDS and provide diagnostic and treatment services to suspects and AIDS patients [57, 61]. PPPs were also used for the provision of diagnostic, treatment, and management of TB in Zambia, Vietnam, Indonesia, South India, Nigeria, Kenya, Nepal, Uganda, Korea, Bangladesh, and South Africa [4, 8, 13, 20, 22, 26, 29, 34, 40, 41, 43, 47, 50, 51, 56, 58, 59, 61, 66]. The following models of partnerships were reported: PPPs, Public-Private Mix (PPM), Public-Private Workplace Partnership (PWP), and Public-NGO Partnership (PNP). PWP and PNP models were used successfully to provide TB services in South Africa. Sites using PWP were reported to have the highest score of all aspects of quality of care (structure, process, and outcomes). PWP and PNP models were similar to solely public providers in terms of process quality, reflecting a very good knowledge of the treatment guidelines among both private and public providers [4]. PPM was used as a strategic initiative to engage all private and public health care providers in the fight against TB, using international health care standards [47, 71]. Unlike PPP, which is based on long-term contracts with risk sharing and decision-making and a high level of collaboration, PPM involves actors from all sectors for non-contractual collaboration with a vertical disease focus [72]. Overall, evidence suggests that the use of various models of partnerships led to an increase in the TB case detection rates and the success of curative services [4, 8, 13, 20, 22, 26, 29, 34, 40, 41, 43, 47, 50, 56, 58, 59, 61, 66]. Among the mechanisms and tools used to achieve this success were the design of referral forms and treatment cards, the implementation of the referral mechanism, the free distribution of medication**,** and the encouragement of patients to complete the course of treatment. Only two studies reported that participation of the private sector in TB control and care resulted in below the optimal level [29] and poor treatment outcomes [47].

### Challenges and recommendations

Despite some positive outcomes and achievements, detailed analysis of studies results showed that partnerships between public and private sectors faced multiple challenges, particularly during the starting and implementation phases. We grouped these challenges into five areas: education, management, human resources, financial resources, and information and technology systems (Table 2).

**Table 2:** Challenges of Public-Private Partnerships in Primary Healthcare

**Education**	
▪ Inadequate education for justification of people to participate in collaborative projects [38, 51]	
▪ Insufficient knowledge of the private about testing and treatment procedures [51, 56, 61, 67]	
**Management**	
▪ Lack of commitment of public and private sector managers and decision-makers [9, 51, 52, 58, 73]	
▪ Lack of clear vision on the way forward [68, 70]	
▪ Non-formulation of strategic direction for PPPs models by the government [70]	
▪ Lack of accountability, poorly defined roles and lack of advisory committees [30, 52]	
▪ The difficulty of coordinating different members, especially in the early stages [47, 66]	
▪ Poor leadership skills [32]	
**Human resource**	
▪ Lack of trust between the public and private sectors^(9, 47, 54, 57, 70)s^	
▪ Disparities in power actors involved in partnership [15, 32]	
▪ Limited access to people willing and able to participate in the private sector [32, 38]	
▪ Lack of capacity of private practitioners and hospitals to undertake non-clinical tasks such as treatment supervision or prompt recording and reporting of required data [51, 61, 68]	
▪ Lack of perceived ownership of the project by partners [42, 61]	
▪ Reported disrespect and distrust of the public sector towards partners from the private sector [9, 67, 70]	
**Financial resources**	
▪ Downsizing of social capital [15]	
▪ Inadequate financial resources [28, 40, 42, 59, 68]	
▪ Sustainability of the PPPs [49]	
▪ Insecure funding [31, 68, 70]	
▪ Lack of trust by private sector partners at the reimbursement system [51, 62]	
▪ The unwillingness of the public sector to use financial incentives for private sector motivation [43, 67]	
▪ Not setting the specified budget for PPP [9]	
**Information and technology systems**	
▪ A weakness of the private sector in the documentation of services provided by the private sector [34, 35, 41, 49, 51, 53, 56, 63]	
▪ Lack of consistent form of interaction between the public and private sectors due to different systems [9, 15, 51, 67]	
▪ Lack of appropriate monitoring and reporting mechanisms [9, 15, 49, 67]	
▪ Lack of clarity in policies regarding the implementation and evaluation of PPPs [15, 46, 70]	
▪ Absence of support systems for supervision and record-keeping for private-sector employees [26, 58]	
▪ Inefficient administrative system [61, 62]	
▪ Weak capacity to collaborate or regulate [54, 57, 68, 70]	
▪ Weakness in implementing regulations [68]	
▪ Inadequate mechanisms to ensure continuity of care [68]	
▪ Information gaps exist between public and private sector [10, 40, 57, 67]	
▪ Absence of external performance assessment of PPPs [10]	
▪ Lack of standard internal monitoring system [10]	
▪ Failure to define an indicator for evaluation of PPPs [8, 9, 11]	
**Others**	
▪ Low efficiency of the private sector in taking care of the poorest sectors of society [15]	
▪ The poor capacity of the public sector to design and manage contracts with private organisations [10, 45, 68]	

*PPP* Public-Private Partnership

In education, among main problems were an inadequate level of knowledge related to testing and treatment procedures and inadequate knowledge for justification of people to participate in collaborative projects by providers [38, 51, 56, 61, 67]. In management, challenges encompassed lack of strategic vision and commitment from various partners, poorly defined roles and expectations, difficulties in member coordination, and a lack of leadership skills [2, 9, 30, 32, 47, 51, 52, 58, 66, 68, 70, 73]. In human resources, reported challenges related to a lack of trust between private and public partners, ownership identity, disparities in power, and lack of capacity to undertake non-clinical tasks by staff in private clinical settings [9, 15, 32, 38, 42, 47, 51, 54, 57, 61, 67, 68, 70]. For financial resources, issues were rooted in inadequate and insecure funding, questions over the long-term sustainability of PPPs, lack of trust in the reimbursement system used by private partners, and not accounting for PPPs in annual budgeting process [9, 15, 28, 31, 40, 42, 43, 49, 51, 59, 62, 67, 68, 70]. For information and technology systems, challenges originated from unclear policies and regulations regarding the implementation and evaluation of PPPs, problems with documentation and record-keeping in private sector providers, a weak capacity to collaborate between sectors or implement regulations, information gap and lack of standardisation, and lack of sufficient monitoring due to lack of defined indicators [8–11, 15, 35, 41, 46, 49, 51, 53, 56, 63, 67, 70]. Additional challenges arose from low efficiency of the private sector in taking care of the poorest strata of the population, as well as a lack of capacity of both sectors to engage with one another [10, 15, 46, 68].

Studies also provided recommendations on how to overcome reported challenges and create effective partnerships (Table 3). For example, in education this can be done by ensuring that only most up-to-date and evidence-based treatment guidelines are used in both sectors, conducting sensitisation workshops based on needs assessment, as well as developing effective information, education and communication strategies for the communities [13, 26, 28, 29, 34, 49, 63, 68]. In management, one could consider to streamline regular communication and coordination between collaborators, encourage commitment and engagement, ensure that there is appropriate legislation that supports the work of PPPs, clarify roles and responsibilities, set realistic goals and objectives, and ensure better coordination of collaboration [8, 9, 13, 35, 39, 46, 49, 53, 58, 61]. In human resources, it is vital to facilitate good communication between all members of PPPs, encourage a positive attitude towards PPPs, bring strong stakeholders into partnerships, and create a culture of respect, appreciation, and trust [9, 31, 47, 51–53, 57, 74, 75]. For financial resources, it is important to introduce financial incentives, ensure funding sustainability, and identify alternate financial suppliers [6, 28, 36, 56, 59, 69, 74]. For information and technology systems, one should consider placing quality assurance mechanisms, building appropriate legislative frameworks, setting up monitoring and documentation systems, using digital tools, and strengthening information systems [6, 9, 13, 15, 20, 54, 59, 61, 63, 75]. To support these efforts, it is important to have some flexibility in PPPs models and complement it by political and community support of PPPs [38, 51, 53, 61, 76].

**Table 3:** Recommendations for effective Public-Private Partnerships in Primary Healthcare

**Education**	
▪ Improving training of the private health practitioners based on the latest treatment guidelines [13, 28, 29, 49, 51, 63]	
▪ Ensuring the knowledge transfer of the most effective, latest and evidence-based treatment guidelines [29, 39, 47, 49]	
▪ Effective information, education and communication strategies in the community [26]	
▪ Conducting sensitisation workshops [67]	
▪ Conducting retraining courses during participation based on needs assessment [6, 34]	
**Management**	
▪ Choosing a strong interface to ensure coordination between partners [8, 9, 46, 47, 51, 57, 61, 67]	
▪ Organising regular meetings to maintain communication and to foster and coordinate plans [9, 47, 62]	
▪ Creating more commitment among partners [13, 21, 26, 47, 49, 51, 53, 58, 61, 62, 64, 70]	
▪ Creation of a national policy document outlining schemes for PPP [61, 70]	
▪ Having a clear delegation of duties and creating process indicators for initiating and sustaining partnerships with private providers [20, 30, 35, 53, 58, 61]	
▪ Determining a shared vision, strong governance and effective management to achieve objectives [21, 35, 39, 53, 66, 70]	
**Human resources**	
▪ Creating good communication between all members of the PPPs [9, 31, 51, 57, 70]	
▪ Taking concerns of all members in initial seriously [51]	
▪ Encouraging positive attitudes towards the PPPs, particularly during the initial stages [51, 53]	
▪ Convincing the private sector that they will benefit from the PPPs [51, 61]	
▪ Creating change thinking among staff [5, 9, 67]	
▪ Attracting strong stakeholders into the partnership [47, 50, 51, 53, 57]	
▪ Facilitating respectful relations in the partnership [9, 57, 70]	
▪ Appreciating the attitude and performance of the staff [31]	
▪ Maintaining transparency between sectors to build trust [9, 31, 32, 47, 52, 66, 70]	
**Financial resources**	
▪ Introducing financial incentives for private sector motivation [4, 32, 56, 69]	
▪ Ensuring availability of appropriate funds [6, 28]	
▪ Taking the determination of sustainable financing for partnership seriously [49, 62]	
▪ Identifying alternate financial suppliers [59]	
**Information and technology systems**	
▪ Ensuring the existence of a quality assurance mechanism and program [6, 13, 20, 54, 59, 70]	
▪ Establishing norms [15, 61]	
▪ Tackling morality and accountability issues [15, 51, 65]	
▪ Building a legislative framework [9, 15, 61, 70]	
▪ Defining operational strategies [8, 15, 27, 58]	
▪ Strengthening supervision of private sector [6, 9, 47, 51, 65]	
▪ Setting a monitoring and documentation system for referrals [9, 13, 63]	
▪ Strengthening the information system [39, 57, 67]	
▪ Determining the appropriate referral structure [9, 61, 67]	
▪ Using digital tools in facilitating project operations and also in ensuring adherence to protocols by both providers and patients [42, 68]	
▪ Ensuring continuity in care for patients by the strength of provider networks and determine policies and program actions [31, 61]	
▪ Establishing procedural requirement in getting the funds released timely for grant/reimbursement to the private partner [53]	
▪ Creating a payment-based financial system [27, 32, 46, 69]	
**Others**	
▪ Ensuring flexibility of the PPPs model to adapt to changing circumstances [28, 51]	
▪ Encouraging political and community support for partnership [43, 53, 61, 68]	

*PPP* Public-Private Partnership

## Discussion

We examined the global experience of PHC provision via PPPs for basic PHC services, health education and promotion programs, and services for infectious diseases. The majority of PPPs projects facilitated education and health promotion initiatives and were used to increase access and to facilitate the provision of prevention and treatment services (i.e., TB, malaria**,** and HIV/AIDS) for certain target groups. The challenges of providing PHC via PPPs were reported primarily for the starting and implementation phases of project execution. Reported challenges and recommendations on how to overcome them fell into one of five areas: education, management, human resources, financial resources, and information systems.

To improve the health care delivery system and to overcome the limitations of financial, technical, and human resources aspects, PPPs should be considered for future health reforms [3, 15, 77]. Governments already see the potential for private sector involvement in improving public health and PHC services delivery [74, 78], as they can bring benefits to the health care system, population health, and can lead to direct and indirect costs savings [79]. They also provide an opportunity for mutual learning between colleagues by stimulating the creation of new knowledge and infrastructure, increase transparency, which can provide greater accountability, public confidence, and result in a higher quality of care [75, 76]. PPPs can lead to improvements in efficiency and effectiveness in service provision and provide a necessary platform for social tests that can enable learning, for example, on how to handle the most unsustainable health problems. However, the opponents of PPPs believe that most PPPs are weak, as developing countries do not have the resources to monitor the quality of provided health services [80, 81]. Private medical providers are also accused of self-centred attitudes and non-interference in public works. However, some doctors working for the private sector might be willing to take part in partnerships to be able to fight TB and provide HIV/AIDS services for the target population together with the public sector employees and other health sector representatives. The role of private doctors also needs to be carefully analysed and should be supported in its processes when assuming responsibility as primary caregivers [61].

A partnership should not be formed unless the public sector is strong enough to ensure that it can provide appropriate training and health care services, monitor the outcomes, and have the ability to engage as a partner in PPPs [51]. Before designing any partnership, clear and achievable public interest goals should be considered. A government structure should then ensure that the goals are in line with the needs of stakeholders in public-private partnerships, and tools and mechanisms to measure progress and success are well-defined [82]. All partners should also be motivated and provided with incentives to ensure active engagement and participation [9, 51]. All individuals who participate in the partnership must have the appropriate level of bargaining power. Hence, to form a common attitude among all partners, sensitisation and persuasion training is also recommended [51, 82]. Another important element is transparent communication and accountability of all partners [30, 32, 81].

PPPs can have a better and more stable performance by improving existing healthcare infrastructure, deploying trained human resources, and, most importantly, by better monitoring doctors and professionals and managed organisations [67]. Although the implementation of a PPP model is not easy, it could be even harder to maintain it [51]. Sustainability of participatory models is one of the important issues. A lack of financial support and commitment, especially at the level of top executives, are among the issues that can distort the model’s sustainability [9, 51, 68]. Hence, the sustainability of each model of PPP depends on the ability, commitment, collaboration, and communication between the public and private sectors [9, 32, 51, 70].

Additionally, long-term planning and sustainability policies should be considered, as well as any additional health care costs. Alternative and sustainable funding sources should be identified, and PPPs must be prepared to respond to possible problems, seize the opportunities, anticipate external threats**,** and be flexible. The weaknesses and deficiencies of any partner involved in PPPs could potentially affect the provision and quality of PHC services. However, ultimately, it is the government and local health authorities that are responsible for PHC services provision to the population [27, 51, 68, 82].

## Limitations

Our study is one of the first to review PHC services provision via PPPs. The key weaknesses of our review should, nonetheless, be kept in mind. First, our findings reflect the results of partnerships in PHC and left studies reporting on PPP use in hospitals and other healthcare sectors outside the scope of this review. Second, we only reviewed studies that were published in the English language, potentially leaving important studies reported and published in other languages.

## Conclusion

Despite various challenges, PPPs could provide a good opportunity to facilitate access to health care services, especially in remote areas. However, it should be noted that the success of PPPs depends on the existence of transparency in relationships between partners, PPPs being flexible, having a sustainable financing source, mutual commitment**,** and the ability of the public sector to monitor and control the quality of services provided by the private sector. Therefore, governments should consider long-term plans and sustainable policies to start such partnerships and learn from the experience of other countries.

## Data Availability

All data generated or analysed during this study are included in this published article and its supplementary information files.

## Appendix

**Table 4:** Appendix: Search strategy

Database	Keywords
Science direct PubMed Embase Web of science	A: PPP OR “public- private partnership” OR “public- private participation” OR “public -private collaboration” OR “public- private engagement” OR “Public- private mix” B: “Health care “OR “Healthcare” OR “public health” C: A AND B
Ovid Medline	A: PPP OR “public- private partnership” OR “public- private participation” OR “public -private collaboration” OR “public- private engagement” OR “Public- private mix” B: PHC OR “Primary Health care “OR “Primary Healthcare” C: A AND B
Scopus	A: PPP OR “public- private partnership” OR “public- private participation” OR “public -private collaboration” OR “public- private engagement” OR “Public- private mix” B: PHC OR “Primary Health care “OR “Primary Healthcare” OR “public health” C: A AND B
Inclusions criteria	All studies on public-private partnerships in primary health care delivery.
Exclusions criteria	Studies published in any language other than English. Non-peer reviewed reports, documents and posters. Unpublished manuscripts.

## Abbreviations

PPP: Public-Private Partnership
PHC: Primary Healthcare
UHC: Universal Health Coverage
PPPs: Public-Private Partnerships
TB: Tuberculosis
PPM: Public-Private Mix
PWP: Public-Private Workplace Partnership
PNP: Public-NGO Partnership
